# Prolonged Neurological and Musculoskeletal Symptoms Following Shingrix Vaccination

**DOI:** 10.3390/reports7040083

**Published:** 2024-10-09

**Authors:** Sabrina Hollar, Amna Khalid, Benjamin D. Brooks, Michael Wons

**Affiliations:** 1 Department of Biomedical Sciences, Rocky Vista University, Parker, CO 80134, USA; sabrina.hollar@co.rvu.edu; 2 Department of Biomedical Sciences, Rocky Vista University, Ivins, UT 84738, USA; amna.khalid@ut.rvu.edu (A.K.); michael.wons@rvu.edu (M.W.); 3 Maine Medical Center, Internal Medicine Department, Portland, ME 04102, USA

**Keywords:** recombinant zoster vaccine, Shingrix, adverse reaction, joint pain, neurological symptoms, long-term symptoms, vaccine safety

## Abstract

**Background and Clinical Significance**: The recombinant zoster vaccine (Shingrix) helps prevent shingles and its complications in adults 50 and older. While minor side effects are common, severe adverse reactions are thought to be rare, and long-term side effects are not well documented. **Case Presentation**: A 50-year-old woman with Crohn’s disease developed joint pain, effusion, and neurological symptoms such as numbness and tingling shortly after receiving the first dose of the recombinant zoster vaccine. Symptoms waxed and waned but persisted for over a year despite anti-inflammatories and specialist referrals. Diagnostic imaging and labs were unrevealing. **Conclusions**: This case of prolonged somatic and neurological symptoms associated temporally with Recombinant zoster vaccine administration reinforces the critical need for thorough pharmacovigilance and investigation of possible long-term adverse vaccine reactions. Provider documentation and reporting of individual experiences can help improve the detection of rare vaccine-related risks, elucidate potential risk factors, and refine safety screening. Diligent monitoring and research into longitudinal vaccine outcomes remain paramount, especially following recent emergency authorizations.

## 1. Introduction and Clinical Significance

The recombinant zoster vaccine (Shingrix) is a two-booster recombinant vaccine released by the FDA in 2017 to prevent shingles (herpes zoster) in patients aged 50 and older who are at risk. The vaccine combines the varicella zoster virus glycoprotein E antigen with the AS01B adjuvant system [1]. Glycoprotein-E antigen is expressed during VSV infection and antibodies are readily detected in adults with prior varicella infection [2]. To enhance cellular immune response, the adjuvant AS01B, which contains QS21, a plant-derived saponin, and monophosphoryl lipid A (MPL), a Toll-like receptor 4 agonist, was added [2]. These components stimulate antibody production and enhance glycoprotein-E-specific cell-mediated immunity [1]. The shingles vaccine is administered as a two-dose series. For most healthy patients the booster is administered 2 to 6 months after the first dose, while immunocompromised patients may receive the booster 1 to 2 months after the first dose [1].

The estimated lifetime incidence of herpes zoster ranges from 10 to 20 percent [3]. Mortality from the condition is rare, with an incidence of between 0 and 0.47 cases per 100,000 individuals being reported annually [3]. Although the mortality for herpes zoster is low, the virus has been attributed to causing a painful vesicular rash [4]. More importantly, the virus has the potential to cause post-herpetic neuralgia, a complication of shingles in 10–18% of affected patients that causes severe neuralgia, which may or may not resolve over time. According to the Centers for Disease Control and Prevention (CDC), the recombinant zoster vaccine is 97% effective in preventing shingles in adults 70 years and older [5]. Among the side effects reported by the CDC, the most common include mild or moderate pain, redness and effusion at the injection site, fatigue, headache, myalgias, fever, abdominal pain, gastrointestinal symptoms, and nausea. In addition, severe symptoms that may develop include an allergic reaction—skin rash, itching, flushing, effusion, and anaphylaxis [5]. Although rare, neuropathy has been recognized as an adverse event following the administration of the Recombinant zoster vaccine [6]. Neurological adverse events following the administration of the vaccine have been thought to be due to to the QS-21 component in the AS01 adjuvant in the recombinant zoster vaccine [7]. Through molecular mimicry from vaccine administration, it is hypothesized that patients experiencing adverse reactions may express immune cross-reactivity, leading to autoimmune-like symptoms [2,8]. Although instances of temporary neuropathy have been reported, this vaccine’s chronic neurological side effects have not been well documented in the Vaccine Adverse Event Reporting System (VAERS) database or been listed by the CDC. 

While the recombinant zoster vaccine has demonstrated efficacy against herpes zoster, documenting and thoroughly investigating any possible long-term adverse symptoms remains critical for ongoing pharmacovigilance and improving patient safety profiles, especially among vulnerable groups. Although research has been carried out on the long-term effects, further studies are essential to enhance patient prognosis, focusing on preventive measures and therapeutic approaches [3]. This case is one of multiple in a series in which a patient developed long-lasting side effects (over six months) after receiving the recombinant zoster vaccine.

## 2. Case Presentation

**Figure 1:**
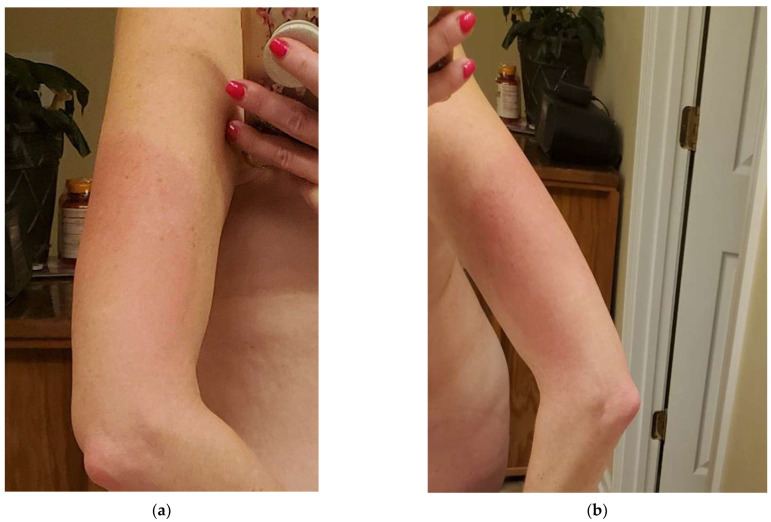
(**a**) Enlarged, warm, and erythematous injection site on the patient’s left deltoid post vaccination day 2 (**b**) An alternate angle of the patient’s left deltoid reaction post vaccination.

**Figure 2:**
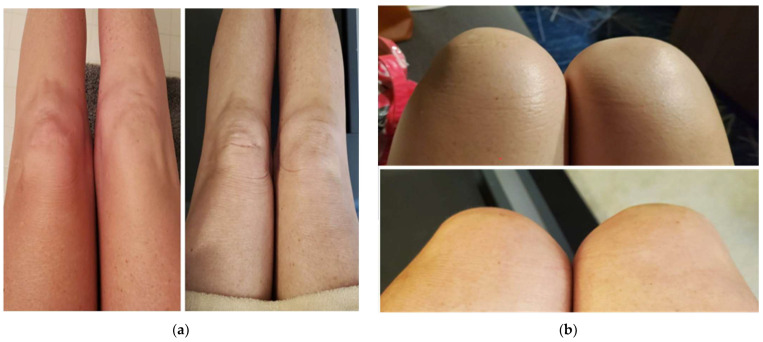

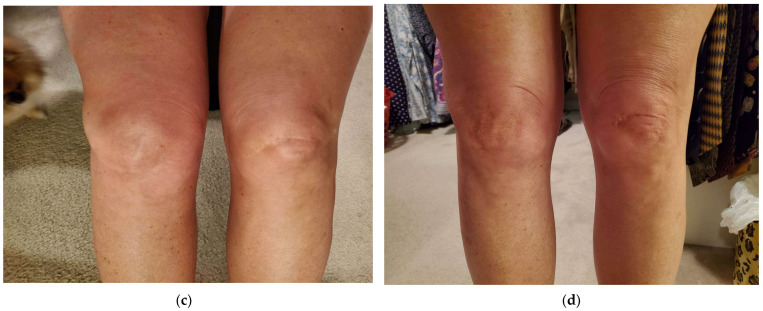
(**a**,**b**) Depictions of the patient’s swollen knees in a seated position (swelling on left, without swelling on the right); (**c**,**d**) a comparison of the patient’s knees when swollen (top picture) vs. without swelling (bottom), with figure (**c**) depicting swollen knees and figure (**d**) depicting knees without swelling.

The patient in this case study is a 50-year-old female with a history of Crohn’s disease and right knee pain secondary to a sports injury who received the recombinant zoster vaccine at her primary care office in October 2022. Several hours after receiving the recombinant zoster vaccine in her left deltoid, the patient developed generalized flu-like symptoms, including fever, general myalgias and arthralgias, and fatigue described as a “worse pain than intense exercising.” The day after her injection (day 2), the patient developed nausea and a decreased appetite, as well as her first incident of a tingling sensation accompanied by a burning sensation bilaterally (L > R) in her arms along with her left anterior thigh. The injection site on the left deltoid was significantly enlarged, warm, and erythematous (see Figure 1 below) and these symptoms lasted approximately two weeks before complete resolution. On day three, her fever, nausea/decreased appetite, myalgias, and fatigue resolved; however, she had lingering pain, described as an intense “ache” in her joints, including her elbows, ankles, and knees bilaterally, along with visible swelling in these affected joints (see Figure 2 below).

For the month of November 2022, the pain and swelling persisted. Symptoms were reported to be worse in the mornings, with her pain rated to be an eight out of ten, slightly improving as the day progressed to a six out of ten. The patient felt that the pain significantly impacted her gait and mobility, limiting her range of motion with daily activities. At this point, the patient contacted her primary care physician, who recommended the use of over-the-counter NSAIDs. Despite daily use as directed, no improvements were noted. Additionally, the patient continued to experience intermittent numbness and tingling, as described in the locations above.

Between November 2022 and January 2023, the patient continued to experience daily pain and swelling in joints without any improvements despite daily OTC ibuprofen. The patient also reported intermittent tingling bilaterally (L > R) in her arms and left lower extremity. The patient spoke with her primary care physician during that time and was referred for a nerve conduction study, which showed the presence of carpal tunnel, otherwise, no significant findings were reported. In January 2023, the patient discontinued daily ibuprofen use and began the application of topical herbal substances—helichrysum and frankincense oil—along with using wraps and kinetic tape. The patient reported mild improvements in her discomfort and effusion with daily application. Following the results, her PCP suggested that she see orthopedics for further evaluation. The patient underwent X-ray imaging in February 2023, which revealed no abnormal findings. The patient’s PCP also ordered labs around that time, which were negative for any abnormalities in her inflammatory markers (see Table 1 below). Repeat labs completed in April 2023 showed no abnormalities in her inflammatory markers.

By May 2023, her joint pain and effusion had gradually improved with the nightly use of topical oils. By now, the patient no longer needed to wrap her joints daily—only as needed. The patient reported recurrent and increasing episodes of pain and effusion in her joints if treatments were missed. The frequency of episodes had significantly improved to once a month compared to daily several months prior.

As of August 2023, the patient continued to experience intermittent tingling in the arms and lateral aspect of the left lower extremity and intermittent joint pain and effusion despite daily herbal oil application. The patient reported seeing multiple primary care providers who could not explain the cause and recurrence of episodes of pain, effusion, and tingling in the areas as described in detail above. After experiencing these symptoms, the patient declined the administration of the second booster as recommended by her current primary care provider with concerns that this may do further harm than good.

## 3. Discussion

Reviewing this complex case highlights several opportunities to optimize care for patients with prolonged, difficult-to-diagnose symptoms following vaccination. For example, while repeated lab work and imaging were appropriately negative in this case, referral to specialists like rheumatology or neurology could have provided additional expert assessment of her joint and neurological symptoms. Enhanced continuity of care with her primary provider may have led to an earlier multidisciplinary approach. The patient reported seeing multiple providers over a year with little continuity of care. The patient also admitted the reluctance of these physicians to accept that the cause of her symptoms was indeed the result of the vaccine.

QS-21, a saponin derived from the Quillaja saponaria tree, is widely used as an adjuvant in various vaccines to enhance immune responses [8]. One of the most notable examples, as already discussed previously, is the recombinant zoster vaccine (Shingrix), where QS-21 is a part of the AS01B adjuvant system. QS-21 is also found in other vaccines like Mosquirix, the malaria vaccine used in regions with a moderate-to-high transmission of *Plasmodium falciparum*, and the NVX-CoV2373 COVID-19 vaccine by Novavax, which uses the “Matrix-M” adjuvant system (containing a mix of QS-21 and other similar compounds, QS-17 and QS-7) [9]. Additionally, QS-21 is being explored in vaccines for diseases such as HIV, cancers, and Alzheimer’s disease, highlighting its flexibility as an adjuvant in both preventive and therapeutic applications [9].

While these vaccines generally show strong safety profiles, some studies suggest that neurological adverse events, particularly with Shingrix, may be linked to the QS-21 component in the AS01 adjuvant [7]. It has been hypothesized that molecular mimicry triggered by the vaccine could lead to immune cross-reactivity, causing autoimmune-like symptoms in certain individuals [2]. Although short-term side effects like temporary reactogenicity have been recorded in all these vaccines, long-term side effects have not been extensively documented in the Vaccine Adverse Event Reporting System (VAERS) or by the CDC. Further research is needed to understand any potential chronic effects.

This case is unique because the patient’s symptoms, as described above, have not been reported among the listed side effects of the recombinant zoster vaccine. The most common adverse events reported in clinical trials were pain at the injection site (78.7%), myalgias (45.4%), fatigue (45%), headache (38.1%), chills (31.9%), fever (30.5%), and gastrointestinal symptoms (26.8%) [3]. More importantly, no long-term side effects have been reported in experienced trials, as evidenced by the continuation of this patient’s symptoms almost one year after her vaccination. While initial safety data on vaccines is under extensive review and obtained via pre-licensure clinical trials and short-term marketing surveillance, ongoing monitoring for adverse events over longer periods of time are crucial to determine their benefits and risks in the patient population. Rare vaccine risks may only become evident after millions of doses have been administered, and some adverse events may emerge months or even years after vaccination. Therefore, robust systems like VAERS are crucial for ongoing safety surveillance. Healthcare providers should closely monitor patients’ health following vaccination and report any events that may raise concern. This requires openness to the possibility of rare vaccine-associated effects, even without established causality. Patient concerns and experiences warrant careful documentation regardless of presumed etiology. Collecting long term data for vaccines such as the recombinant zoster vaccine may provide a further understanding of their safety profile. Increased reporting of adverse events can also strengthen post-marketing surveillance to refine usage guidelines and identify high-risk populations that may benefit from more tailored vaccine recommendations. More studies are also needed to determine the causal relationship between patient populations who are susceptible to developing long-term adverse side effects after receiving the recombinant zoster vaccine. Going forward, this case reinforces the need for further long-term pharmacovigilance and studies to better characterize the incidence of long-term side effects after administration of the recombinant zoster vaccine, especially in vulnerable subgroups.

Although we have discussed the possibility of adverse events, including neuropathy associated with the recombinant zoster vaccine, the morbidity and mortality of shingles in unvaccinated individuals remain significantly more substantial. Each year, approximately 1 million Americans are affected by shingles. Complications, including post-herpetic neuralgia, occur in roughly 13–40% of shingles cases. Shingles can cause extreme acute pain, resulting in significant negative impacts to quality of life. In some instances, post-herpetic pain is a lifelong complication requiring pain medication [4]. Despite concerns regarding post-vaccination side effects, the incidence of vaccine side effects is generally low. The recombinant zoster vaccine reduces shingles risk by 97% and lessen complications for individuals who do develop shingles post-vaccination [1]. As a result, we can conclude that the benefits far outweigh the risks. Close monitoring for adverse events and comparisons to shingles morbidity and mortality are crucial, but currently the benefits of vaccination outweigh the potential harms. 

It is crucial to emphasize that this case report, which highlights a concerning adverse event following the administration of the recombinant zoster vaccination, should not be interpreted as anti-vaccine literature. Documenting and comprehending safety signals, even those that are rare, enables proper education and a well-informed assessment of benefits and risks. Extensive data overwhelmingly support the significant public health advantages of the Recombinant zoster vaccine for appropriate populations [10]. Nevertheless, maintaining transparency regarding complete safety profiles is equally essential. We caution against drawing broad conclusions or exaggerating implications based on limited case reports. Healthcare providers should continue to recommend this vaccine in accordance with guidelines. Monitoring for potential risks does not diminish vaccine confidence or effectiveness.

## 4. Conclusions

This case serves as a reminder of the imperative for continued monitoring and research into longitudinal vaccine outcomes. The persistence of somatic and neurological symptoms in this patient, despite standard interventions and negative diagnostic findings, highlights the need for heightened awareness among healthcare providers regarding the possibility of vaccine-related complications. Documentation and reporting of individual experiences, as exemplified in this case, can facilitate the early detection of rare adverse events and contribute to refining safety screening protocols to safeguard vulnerable patient populations. Enhanced reporting mechanisms and interdisciplinary collaboration can further strengthen post-marketing surveillance, ultimately optimizing patient safety and informing tailored vaccine recommendations. We emphasize the importance of raising awareness and maintaining vigilance regarding rare but serious vaccine-related adverse events. We encourage the use of VAERS, along with strong patient-provider communication and education, to enhance outcomes and expedite diagnosis.

## Figures and Tables

**Table 1:** Lab results ordered on 6 February 2023 and completed on 10 February 2023; results reviewed on 12 February 2023.

Labs	Values	Reference Ranges
HgbA1c	5.0%	<5.7%
T3, Reverse	21	8–25 ng/mL
T3, free	4.9 (H)	2.3–4.2 ng/mL
T3 (TT3)	179 (H)	60–170 ng/dL
Free T4	1.1	0.8–1.8
Vitamin B-12	>2000 (H)	180–914 pg/mL
Methylmalonic acid	166	87–318 nmol/L
Homocysteine	7.7	<10.4 μmol/L
Magnesium	2.0	1.7–2.8 mg/dL
Magnesium, RBC	5.4	4.0–6.4 mg/dL
C-reactive protein	<0.40	Normal < 1.0
Anachoice(R) screen	Negative	Negative
Iron	139	26–154 UG/DL
UIBC	201	162–408 UG/DL
Tibc	340	259–492 UG/DL
Transferrin saturation	41% (H)	8.9–40.5%
Ferritin	156.9 (H)	13–150 ng/mL
Vitamin D, hydroxy	107 (H)	30–100 ng/mL
Rheumatoid Factor	<14	<14 IU/mL
CCP Ab IgG	<16	Negative: <20 Weak positive: 21–39 Moderate positive: 40–59 Strong positive: >59

## Data Availability

The data presented in this study are available on request from the corresponding author. The data are not publicly available due to privacy concerns.
